# Neuroprotective Roles of the Reverse Transsulfuration Pathway in Alzheimer’s Disease

**DOI:** 10.3389/fnagi.2021.659402

**Published:** 2021-03-16

**Authors:** Bindu Diana Paul

**Affiliations:** The Solomon H. Snyder Department of Neuroscience, Johns Hopkins University School of Medicine, Baltimore, MD, United States

**Keywords:** Alzheimer’s disease, hydrogen sulfide, cysteine, redox, sulfhydration/persulfidation, transsulfuration

## Abstract

The reverse transsulfuration pathway has emerged as a central hub that integrates the metabolism of sulfur-containing amino acids and redox homeostasis. Transsulfuration involves the transfer of sulfur from homocysteine to cysteine. Cysteine serves as the precursor for several sulfur-containing molecules, which play diverse roles in cellular processes. Recent evidence shows that disruption of the flux through the pathway has deleterious consequences. In this review article, I will discuss the actions and regulation of the reverse transsulfuration pathway and its links to other metabolic pathways, which are disrupted in Alzheimer’s disease (AD). The potential nodes of therapeutic intervention are also discussed, which may pave the way for the development of novel treatments.

## Introduction

Alzheimer’s disease (AD) is a relentless, progressive neurodegenerative disorder predominantly affecting the hippocampus and the cortex leading to memory loss, cognitive deficits, and impaired executive function (Masters et al., 2015; Lane et al., 2018). AD is the most common form of dementia that afflicts 10–30% of the population 65 years or older, resulting in late-onset AD (LOAD) (Masters et al., 2015; Long and Holtzman, 2019; Alzheimer’s Association, 2020). AD may either be familial or sporadic, with either early onset (EOAD) or late-onset (LOAD) with familial causes constituting less than 5% of the total cases (Karch and Goate, 2015). The disease has a long prodromal and preclinical phase, and it may take years for symptoms to manifest, the most noticeable of which include memory impairments and language difficulties (Bateman et al., 2012; Villemagne et al., 2013; Vermunt et al., 2019). At the pathological level, AD is characterized by the accumulation of amyloid plaques, neurofibrillary tangles (NFTs), and paired helical filaments (PHFs) (Glenner and Wong, 1984; Masters et al., 1985, 2015; Grundke-Iqbal et al., 1986; Kosik et al., 1986; Wood et al., 1986).

Regardless of the cause of AD, elevated oxidative stress has been observed in AD, which contributes to disease progression (Torres et al., 2011; Martínez de Toda et al., 2019; Arslan et al., 2020). Oxidative stress refers to an imbalance in redox signaling, where physiological levels of reactive oxygen species (ROS) mediate signal transduction processes (oxidative eustress) and excess has deleterious effects (oxidative distress) (Sies et al., 2017). In AD, Aβ_1–42_, which is generated by the cleavage of amyloid precursor protein (APP), by enzymes such as β-secretase 1 (BACE1) and increases oxidative stress. Aβ_1–42_ elevates oxidative stress by increasing the formation of Fe^2+^ and Cu^+^, which generate hydroxyl radicals (^•^OH) *via* the Fenton reaction, which elicits oxidative damage (Imlay et al., 1988; Huang et al., 1999a,b). Additionally, Aβ_1–42_ activates the Jun N-terminal kinase (JNK) pathway, which is linked to upregulation of BACE1, which mediates increased cleavage of APP to produce more Aβ_1–42_, resulting in a vicious feed-forward cycle (Yao et al., 2005; Guglielmotto et al., 2011). A point to be noted is that Aβ peptides can act as antioxidants as well (Kontush, 2001). It has been proposed that the accumulation of Aβ peptides is protective (Smith et al., 2002). At lower concentrations, in the range of 0.1–1.0 nm, in body fluids, Aβ peptides prevent autooxidation of lipoproteins and plasma low-density lipoprotein (LDL) in the cerebrospinal fluid (CSF). Similar to Aβ_1–40_, the Aβ_1–42_ peptide is also capable of exerting antioxidant effects and the antioxidant activity has been attributed to its metal-chelating effect. At higher concentrations, however, its antioxidant action was abolished (Kontush et al., 2001).

Chronic oxidative stress also affects Tau to elicit neurotoxicity. Tau is hyperphosphorylated in response to oxidative stress, which leads to the formation of NFTs and neurotoxicity (Zhu et al., 2005; Su et al., 2010). Several studies report that oxidative stress may precede symptoms and pathology of AD (Nunomura et al., 2001; Zhu et al., 2004). Oxidative stress in AD is also linked to mitochondrial dysfunction, inflammation, and hypoxia, and these processes culminate in vicious cycles that contribute to neurodegeneration (Bonda et al., 2014; Oliver and Reddy, 2019; Merelli et al., 2020; Butterfield and Boyd-Kimball, 2020).

**Figure 1:**
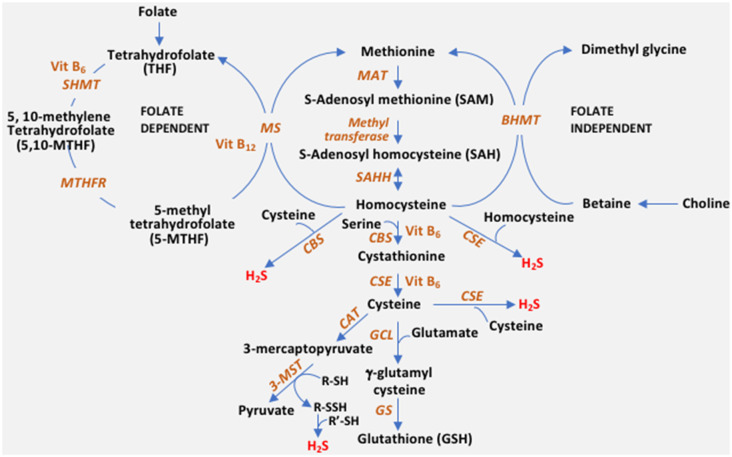
The reverse transsulfuration pathway and inputs from associated pathways. Transsulfuration refers to the transfer of sulfur from homocysteine to cysteine. Dietary methionine is converted to homocysteine, *via* S-adenosylmethionine (SAM) and S-adenosylhomocysteine (SAH). Homocysteine is condensed with serine by cystathionine β-synthase (CBS) to form cystathionine, which is utilized by cystathionine γ-lyase (CSE) to generate cysteine. Cysteine can either enter the glutathione (GSH) biosynthetic pathway or be utilized as a substrate for hydrogen sulfide (H_2_S) biosynthesis. CBS generates H_2_S efficiently from a combination of cysteine and homocysteine, whereas CSE can utilize either cysteine or homocysteine by itself to generate the gaseous signaling molecule. A third enzyme, 3-mercaptopyruvate sulfurtransferase (3-MST) also generates H_2_S. 3-MST utilizes the 3-mercaptopyruvate generated by cysteine aminotransferase (CAT) by forming a persulfide on its active site (R-SH to R-SSH). The persulfide releases H_2_S in the presence of a reductant (R’-SH). Cysteine is converted to GSH by the sequential action of glutamyl cysteine ligase (GCL) and glutathione synthase (GS). Homocysteine is at the junction of the transsulfuration and remethylation pathway (where methionine is regenerated from homocysteine). Remethylation of homocysteine occurs in both a folate-independent and dependent pathway. In the folate-independent pathway, betaine/trimethylglycine (derived from choline) donates methyl groups in a reaction catalyzed by betaine–homocysteine methyltransferase (BHMT) to generate methionine. In the folate-dependent pathway, the vitamin B_12_-dependent enzyme, methionine synthase (MS), converts homocysteine to methionine and tetrahydrofolate (THF), where 5-Methyltetrahydrofolate (5-MTHF) acts as the methyl group donor. THF is converted to 5,10-methylenetetrahydrofolate (5,10-MTHF) by serine hydroxymethyltransferase (SHMT) which utilizes serine and vitamin B_6_. 5,10-methylenetetrahydrofolate reductase (MTHFR), then reduces 5,10-MTHF to 5-MTHF, which remethylates another molecule of homocysteine. MTHFR uses flavin adenine dinucleotide (FAD; the active form of vitamin B_2_) as a cofactor.

The reverse transsulfuration pathway plays a pivotal role in the maintenance of redox balance in cells. Transsulfuration involves the transfer of sulfur from homocysteine to form cysteine *via* cystathionine with connections to the transmethylation pathway and one-carbon metabolism (Figure 1). In addition to serving as a building block for protein synthesis, cysteine is channeled into multiple pathways to generate sulfur-containing molecules including glutathione, the cellular antioxidant, taurine, coenzyme A and lanthionine (Paul et al., 2018). The availability of cysteine is the rate-limiting step for the synthesis of glutathione (GSH) in cells, which maintains redox homeostasis. Diversion of cysteine from protein synthesis to synthesize glutathione is neuroprotective (Ratan et al., 1994). Also, cysteine is the substrate for the generation of the gaseous signaling molecule, hydrogen sulfide (H_2_S). Three enzymes, cystathionine γ-lyase (CSE), cystathionine β-synthase (CBS), and 3-mercaptopyruvate sulfurtransferase (3-MST) generate H_2_S in cells. Besides, CSE and CBS may also utilize homocysteine to generate H_2_S (Paul and Snyder, 2012; Paul et al., 2018). H_2_S, like nitric oxide (NO), modulates a myriad of cellular processes, including transcriptional regulation, response to stress, and mitochondrial function (Krishnan et al., 2011; Paul et al., 2018, 2020; Sbodio et al., 2018). Dysregulated transsulfuration has been observed in several pathological conditions and during aging. In this review, we focus on the status of the transsulfuration pathway in Alzheimer’s disease.

## Elevated Homocysteine, Dementia, and Ad

Homocysteine is at the intersection of the transsulfuration and transmethylation pathway (Figure 1). Homocysteine may either be remethylated to methionine or utilized by the transsulfuration pathway to form cysteine. Hyperhomocysteinemia (elevated levels of homocysteine in the blood) is a risk factor for the development of cardiovascular disease and neurodegenerative diseases including dementia, AD, and Parkinson’s disease (PD) (Seshadri et al., 2002). Elevated homocysteine has also been observed in cases of epilepsy and neuropsychiatric disorders (Herrmann et al., 2007). The involvement of homocysteine in dementia and AD was noted more than 20 years ago using clinical and histochemical analyses (Clarke et al., 1998; Smith et al., 2018). The levels of homocysteine have been correlated with the severity of AD in several studies (Kitzlerová et al., 2014; Farina et al., 2017). Hyperhomocysteinemia has been linked to irreversible neurological deficits and could stem from deficits in levels of folate, vitamin B_6,_ or vitamin B_12_ (Clarke et al., 1998; Smith and Refsum, 2016). Homocysteine mediates toxicity in multiple ways, all of which lead to conditions that pose a risk for developing AD. Homocysteine may elicit neurotoxicity by activation of the *N*-methyl D aspartate (NMDA) receptors to cause excitotoxicity, acting as an agonist at the glutamate binding site as well as a partial antagonist of the glycine co-agonist site (Lipton et al., 1997). Overactivation of the NMDA receptor has been reported to mediate brain damage in focal ischemia (Simon et al., 1984; Lipton and Rosenberg, 1994). Overstimulation of the NMDA receptor increases calcium influx leading to an imbalance in excitatory-inhibitory neurotransmission in the hippocampus of the brain and altered extracellular levels of neuroexcitatory (aspartate) and neuroinhibitory (GABA) neurotransmitters leading to excitotoxic neuronal death and seizures. Homocysteine also causes aberrant processing of the amyloid precursor protein (APP) by a mechanism involving hypomethylation (Lin et al., 2009). Additionally, homocysteine elicits the DNA damage response in neurons and promotes apoptosis and hypersensitivity to excitotoxicity (Kruman et al., 2000). Similarly, increased homocysteine levels were linked to DNA damage in patients deficient in CBS (Vanzin et al., 2014). Elevated homocysteine levels have been linked to traumatic brain injury (TBI) as well, which confers a greater risk for developing AD than stroke (Rahmani et al., 2016). Thus, homocysteine may not only mediate vascular injury, leading to stroke (a risk factor for AD), but also perpetuate the downstream neurotoxic responses. Conversion of homocysteine to homocysteic acid (HCA), a potent glutamate analog, and cysteine sulfinic acid (CSA), which are excitotoxins and generators of oxidative stress, also lead to neuronal damage (Bleich et al., 2000; Obeid and Herrmann, 2006).

### Causes of Hyperhomocysteinemia

Homocysteine can be consumed by either its conversion to methionine by the transmethylation pathway or by its conversion to cysteine *via* the reverse transsulfuration pathway (Figure 1). Since dietary folate and vitamin B_12_ influence the channeling of homocysteine into the reverse transsulfuration pathway, levels of these cofactors may modulate H_2_S production. Decreased expression or activities of the enzymes methylenetetrahydrofolate reductase (MTHFR), methionine synthase (MS), CBS, or CSE may lead to hyperhomocysteinemia. MTHFR is the rate-limiting step in the methyl cycle, catalyzing the conversion of 5,10-methylenetetrahydrofolate to 5-methyltetrahydrofolate, a methyl donor for remethylation of homocysteine to methionine by MS, using NADPH as the reducing agent (Figure 1). MTHFR is the rate-limiting enzyme catalyzing folate production. Mutations in the *MTHFR* gene, which reduce its activity, confer a risk factor for developing AD and atherosclerosis. The mutations that elevate homocysteine include 677C > T, 1298A > C (Castro et al., 2003). The levels of homocysteine in the serum of AD patients and those with mild cognitive impairment (MCI) have been reported to be significantly higher than normal subjects (Joosten et al., 1997; Clarke et al., 1998; Kim et al., 2013; Ma et al., 2017). Similarly, mutations in *CTH*, the gene encoding CSE, are also linked to elevated homocysteine levels, for instance, the 1364G>T (Ser403Ile) and *CTH* 1364T/T homozygotes were associated with hyperhomocysteinemia (Wang and Hegele, 2003; Wang et al., 2004). The first report of *CTH* mutations in cystathioninuric individuals reported two frameshift mutations, c.940_941delCT (p.Leu262ThrfsX20), and c.1220delC (p.Thr355IlefsX18) and two missense mutations, c.200C>T (p.Thr67Ile) and c.718C>G (p.Gln240Glu) (Wang and Hegele, 2003). The single nucleotide polymorphism in *CTH*, originally described as c.1364G>T (p.S403Ile) was renamed as.c.1208G>T (p.Ser403Ile), according to the current convention (Kraus et al., 2009). Consistent with these observations, mice lacking CSE and CBS display increased homocysteine levels in both the blood and the CSF (Yang et al., 2008; Akahoshi et al., 2019). Increased homocysteine concentration compromises the integrity of the blood-brain barrier in mice as measured in the *Cbs*^+/–^ mice (Kamath et al., 2006).

Various strategies have been utilized to counter the toxicity induced by elevated homocysteine levels, including supplementation with vitamin B_6_. Administering B_6_ (pyridoxine) may be effective where defective transsulfuration occurs, as the enzymes CSE and CBS utilize pyridoxal 5- phosphate (PLP) as a cofactor, which influences the flux through the reverse transsulfuration pathway (Lima et al., 2006; Gregory et al., 2016). Interestingly, in Huntington’s disease (HD), impaired PLP metabolism has been observed both in mouse models as well as in patients, which together with the impaired transsulfuration pathway, contributes to impaired cysteine and H_2_S metabolism (Sbodio et al., 2016; Sorolla et al., 2016). In several instances, supplementation with betaine has proven beneficial as betaine serves as the methyl donor in the remethylation of homocysteine to methionine and S-adenosylmethionine (SAM) (Chai et al., 2013; McBreairty et al., 2016). In cases, where mutations occur in the *MTHFR* gene, this strategy may not work. Vitamin B_12_ therapy has also proven beneficial in delaying symptoms of AD. Vitamin B_12_ (cobalamin) serves as a cofactor for methionine synthase, which is one of the enzymes involved in the conversion of homocysteine to methionine. Methylmalonyl-CoA mutase (MUT) is the only other enzyme identified, which utilizes vitamin B_12_ (Watkins and Rosenblatt, 1989; Froese et al., 2019). For cobalamin to be utilized, its efficient transit through the intracellular lysosomal compartment and subsequent delivery to the cytosol and mitochondria is required. Lysosomal function is derailed in Alzheimer’s disease (AD) and its utilization is compromised (Zhao et al., 2015).

In a randomized placebo-controlled trial in older men, supplementation with folate, vitamin B_6_ and vitamin B_12_ daily for 2 years decreased homocysteine levels and reduced the rate of increase in circulating levels of amyloid-β1–40 (Flicker et al., 2008). Similarly, folate supplementation for 3 years improved measures of cognitive function in men and women aged 50–70 years with raised plasma total homocysteine and normal serum vitamin B_12_ (Durga et al., 2007). Other studies reported increases in homocysteine levels in a cohort with mild to moderate AD as a function of disease progression, however, no significant decline in either dietary intake or blood levels of vitamin B_12_/folate was observed indicating that other reasons for hyperhomocysteinemia may operate (Farina et al., 2017).

### Suboptimal Activity of the Reverse Transsulfuration Pathway

The reverse transsulfuration pathway leads to the production of cysteine, the availability of which is the rate-limiting step for glutathione (GSH) biosynthesis. Glutathione levels, as measured by *in vivo* proton magnetic resonance spectroscopy, were lower in hippocampi and frontal cortices of patients with AD compared to healthy controls where decreases in glutathione were correlated to decline in cognitive function. Cysteine is taken up by neurons by EAAC1/EAAT3, which exchanges cysteine for glutamate. Soluble Aβ can impair cysteine and glutathione metabolism in cells by inhibiting EAAT3 (Hodgson et al., 2013). Also, EAAT3 accumulates in the detergent-insoluble fraction of hippocampal neurons instead of its normal localization at the plasma membrane and its depletion causes age-dependent neurodegeneration (Aoyama et al., 2006; Duerson et al., 2009). Dysregulated cysteine metabolism has been observed in aging as well as neurodegenerative diseases such as Huntington’s disease (Dedeoglu et al., 2004; Paul et al., 2014; Zivanovic et al., 2019).

## Glutathione Metabolism in Ad

In the APPTg2576 mouse model of AD, a decrease in GSH was observed at 19 months in the cortex of the brain (Dedeoglu et al., 2004). Similarly, in a mouse model of AD B6.Cg-Tg (APPSwe, PSEN1dE9), the ratio of reduced GSH to oxidized GSH (GSSG) decreased as a function of age (Zhang et al., 2012). *In vivo* proton, magnetic resonance spectroscopy studies revealed that GSH levels were decreased in the hippocampi and frontal cortices of patients with AD as compared to normal subjects and a decrease in glutathione was correlated to a decline in cognitive function (Mandal et al., 2015). A separate study reported sex-specific differences in GSH levels, where males with AD exhibited a decrease in blood cells as compared to their normal controls while samples from female subjects displayed no alterations in GSH content (Liu et al., 2005). A decrease in GSH and GSH/GSSG ratio was also observed in the plasma of patients with MCI and AD (Bermejo et al., 2008). Analysis of postmortem samples also revealed diminished GSH levels in mitochondrial, and synaptosomal fractions derived from the frontal cortices of MCI, mild and severe AD cases as compared to controls (Ansari and Scheff, 2010). Based on *in vivo* MRS studies, it was concluded that while hippocampal GSH levels could distinguish between MCI and elderly healthy controls with 87.5% sensitivity, 100% specificity, cortical GSH levels could differentiate MCI and AD with 91.7% sensitivity, 100% specificity (Mandal et al., 2012) and accordingly GSH has been proposed as a marker as well as therapy for AD (Mandal et al., 2019).

## Signaling Mediated by Hydrogen Sulfide

In addition to its essential role as a building block in protein synthesis, cysteine is also the precursor of the gaseous signaling molecule, H_2_S, which participates in a multitude of physiological processes (Wang, 2012; Paul and Snyder, 2015). Three enzymes produce H_2_S endogenously. CSE utilizes cysteine to produce H_2_S, pyruvate, and ammonia (Stipanuk and Beck, 1982; Paul and Snyder, 2012, 2015). CBS condenses cysteine and homocysteine to produce H_2_S in addition to cystathionine (Chen et al., 2004; Paul and Snyder, 2012). 3-MST generates H_2_S in concert with cysteine aminotransferase (CAT). CAT metabolizes cysteine and α-ketoglutarate to form 3-mercaptopyruvate (3-MP). 3-MST acts on 3-MP formed to generate H_2_S and pyruvate. 3-MST also generates H_2_S using D-cysteine as a substrate in conjunction with D-amino acid oxidase (Shibuya et al., 2013). Over 150 mutations in the CBS protein have been reported, several of which cause its misfolding, and are linked to enzyme activity (Kozich et al., 2010). CBS and 3-MST occur mostly in the central nervous system, although these enzymes are also present in peripheral tissues (Shibuya et al., 2009). Similarly, CSE too is present in the brain. Accumulating studies have reported distinct spatial compartmentalization of the three enzymes in the central nervous system. While CBS is predominantly localized to astrocytes and glia, CSE is present in neurons (Enokido et al., 2005; Linden et al., 2008; Morikawa et al., 2012). 3-MST is also present in neurons and acts in conjunction with CAT to produce H_2_S. While CSE and CBS are predominantly cytosolic during basal conditions, they may translocate to the mitochondria or nucleus during stress (Paul et al., 2020). 3-MST is present in both the cytosolic and mitochondrial compartments.

H_2_S exerts its effects through a posttranslational modification termed persulfidation/sulfhydration, involving the conversion of -SH groups to persulfide or -SSH group (Mustafa et al., 2009; Paul and Snyder, 2012; Zivanovic et al., 2019). H_2_S and sulfhydration modulate several homeostatic processes in the central nervous system and its disruption occurs in several neurodegenerative diseases including Parkinson’s disease (PD) and HD (Vandiver et al., 2013; Paul et al., 2014; Paul and Snyder, 2018). H_2_S plays central role in processes regulating cognitive function and neuromodulation. The first study on the role of H_2_S in processes involved in learning and memory demonstrated that physiological concentrations of H_2_S selectively enhance NMDA receptor-mediated responses to modulate the induction of hippocampal long-term potentiation (LTP; Abe and Kimura, 1996). Clinical studies have reported a decrease in H_2_S levels in the plasma of AD patients, which correlated with the degree of cognitive decline (Liu et al., 2008). Additionally, S-adenosyl methionine (SAM), which allosterically activates CBS is depleted in the human AD brain as well as in the cerebrospinal fluid (Morrison et al., 1996; Linnebank et al., 2010). Dietary supplementation with SAM delayed amyloid plaque and Tau pathology in the 3xTg-AD mouse model of AD (Lee et al., 2012). Injection of Aβ_1–42_ into the hippocampi of rats induced cognitive impairment and reduction in H_2_S levels and expression of endogenous CBS and 3-MST and intraperitoneal injection of the H_2_S donor, NaSH, ameliorated cognitive deficits as well as neuroinflammation (Liu et al., 2015). Similarly, several studies have reported the protective effects of H_2_S in mouse models of AD, although the effects of sulfhydration on signaling pathways were not evaluated (Giuliani et al., 2013; Zhao et al., 2016; Vandini et al., 2019).

**Figure 2:**
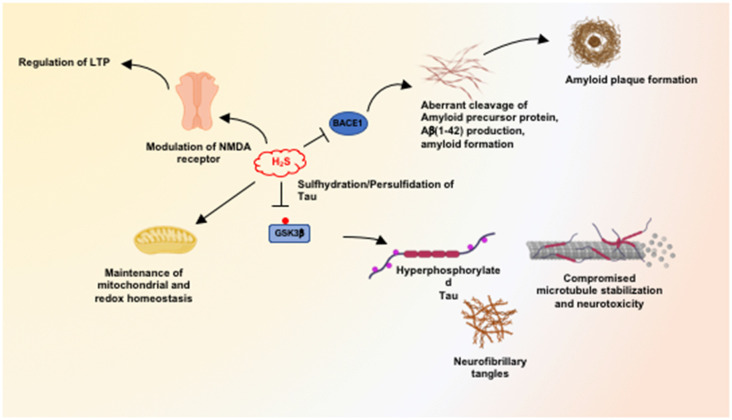
Neuroprotective processes influenced by hydrogen sulfide (H_2_S). H_2_S mediates neuroprotection by modulating multiple pathways, including maintenance of mitochondrial function and redox balance in the brain. At physiological concentrations, H_2_S facilitates long-term potentiation (LTP) in a manner dependent on the *N-*methyl D-aspartate (NMDA) receptors. H_2_S inhibits the expression and activity of beta-secretase 1 (BACE1) and thus inhibits amyloidogenic processing of the amyloid precursor protein (APP) to prevent the accumulation of β-amyloid plaques. H_2_S also mediates persulfidation/sulfhydration of glycogen synthase kinase 3β (GSK3β) and inhibits activity, preventing hyperphosphorylation of Tau which would prevent its aggregation into neurofibrillary tangles (NFTs).

We have shown that H_2_S signaling is dysregulated in AD, and sulfhydration is diminished in the 3xTg-AD mouse model of AD as well as in human AD (Giovinazzo et al., 2021). The protein Tau, which is hyperphosphorylated and aggregated in Alzheimer’s disease, binds CSE and stimulates its catalytic activity. Interestingly, CSE does not bind mutant P301L Tau, which is present in the 3xTg-AD model of AD. Additionally, H_2_S produced by CSE sulfhydrates glycogen synthase kinase 3β (GSK3β) and inhibits its catalytic activity (Figure 2). Sulfhydration of GSK3β was decreased in the cortex of postmortem human AD samples as well. Finally, supplementing NaGYY, the sodium salt of GYY4137, a slow-releasing H_2_S donor, restored sulfhydration and ameliorated motor and cognitive deficits in the 3xTg-AD mice (Giovinazzo et al., 2021).

## Therapeutic Avenues

Analysis of clinical trials conducted so far, reveal a preference for non-amyloid targets, which include therapeutics targeting inflammation, synapse and neuronal protection, vascular factors, neurogenesis, and epigenetic modifications (Cummings et al., 2014). Additionally, there has been an increased interest in repurposed drugs. Several clinical trials involving the use of antioxidants have largely failed (Kim et al., 2015). Some of the antioxidants used only target specific reactive oxygen species (ROS) and thus do not affect other species. Others inhibit physiological processes such as autophagy, causing undesirable side effects (Underwood et al., 2010). Similarly, the timing and duration of intervention could also affect outcomes, as well as patient selection criteria. Thus, therapies which target multiple pathways without compromising normal cellular processes would be more effective. As the gasotransmitter H_2_S regulates a wide array of neuroprotective processes, therapies, and interventions involving either the use of H_2_S delivering drugs or agents which stimulate its production are gaining importance. In mice treated with homocysteine, supplementation of H_2_S donors ameliorated homocysteine-induced cerebrovascular pathology, cognitive deficits, and toxicity (Kamat et al., 2016). Supplementation of H_2_S donors was also reported to be beneficial in several mouse models of AD. Administering sodium hydrosulfide (NaSH), at 2.8 mg/kg, once a day for 3 months ameliorated memory deficits, reduced APP and BACE1, upregulated the master regulator of antioxidant response genes, nuclear factor erythroid-2-related factor 2 (Nrf2), heme oxygenase-1(HO-1) and glutathione S-transferase (GST) in the APP/PS1 model of AD (Liu et al., 2016). The mitochondria-targeted H_2_S donor, AP-39, maintained cellular bioenergetics and preserved mitochondrial function in the APP/PS1 mouse model of AD (Zhao et al., 2016). Other modes of action of H_2_S donors were also reported. For instance, NaSH afforded benefits in the APP/PS1 mice by acting *via* the GluN2B subunit of the NMDA receptor (Yang et al., 2016). Physiological concentrations of H_2_S also increased levels of the second messenger, cAMP, in primary cultures of brain cells, neuronal and glial cell lines, and *Xenopus* oocytes and activated the NMDA receptor in a protein kinase A (PKA)-dependent manner (Kimura, 2000). Thus, H_2_S modulates several neuroprotective pathways, and modulation of its production may be beneficial.

Future areas of research in this area would involve tunable delivery of H_2_S donors so that its concentration in cells and tissues can be modulated.

## Author Contributions

BP conceptualized and wrote the review and prepared the figures.

## Conflict of Interest

The author declares that the research was conducted in the absence of any commercial or financial relationships that could be construed as a potential conflict of interest.
